# The Prognostic Role of the Neutrophil-to-Lymphocytes Ratio in the Most Frequent Cardiovascular Diseases: An Update

**DOI:** 10.3390/life14080985

**Published:** 2024-08-07

**Authors:** Evelina Maria Gosav, Daniela Maria Tanase, Oana Nicoleta Buliga-Finis, Ioana-Irina Rezuș, Paula Cristina Morariu, Mariana Floria, Ciprian Rezus

**Affiliations:** 1 Department of Internal Medicine, “Grigore T. Popa” University of Medicine and Pharmacy, 700115 Iasi, Romania; dr.evelinagosav@gmail.com (E.M.G.); oana_finish@yahoo.com (O.N.B.-F.); adascalitei.paula-cristina@d.umfiasi.ro (P.C.M.); ciprianrezus@yahoo.com (C.R.); 2 Internal Medicine Clinic, “St. Spiridon” County Clinical Emergency Hospital, 700111 Iasi, Romania; 3 Department of Radiology, “Grigore T. Popa” University of Medicine and Pharmacy, 700115 Iasi, Romania; rezus_ioana-irina@d.umfiasi.ro; 4 Radiology Clinic, “St. Spiridon” County Clinical Emergency Hospital, Bulevardul Independentei 1, 700111 Iasi, Romania

**Keywords:** neutrophil-to-lymphocytes ratio, neutrophils, lymphocytes, inflammation, cardiovascular disease, biomarkers

## Abstract

Given the continuous changes in the world, with an increasing trend of unhealthy lifestyles, metabolic comorbidities, and increased susceptibility to cardiovascular diseases (CVDs), researchers change their attention to improve not only the therapeutic platform but also current CVD predictive and prognostic tools to improve disease outcomes. As CVD is characterized by an inflammatory paradigm involving, to some degree, the innate and adaptative immune systems, the neutrophil-to-lymphocyte ratio (NLR) emerged as a potential low-cost, rapidly available, and reliable inflammatory marker, with substantial recent evidence showing its potential utility in clinical practice. Thus, in this literature review, we will present an up-to-date discussion of the prognostic role of NLR in the most frequent CVDs, such as acute and chronic coronary disease, atherosclerotic disease, heart failure, cardiac valvopathies, and cardiac arrhythmias with predilection to atrial fibrillation.

## 1. Introduction

Despite recent therapeutic advances in this field, cardiovascular diseases (CVDs) prevail as the number one cause of death worldwide, being arguably, at the moment, one of the major global health problems. In the face of global negative changes regarding raised environmental risk exposure and unhealthy lifestyles, researchers focus not only on new therapeutic approaches but also on identifying novel CVD biomarkers [1]. Since the discovery of inflammatory cells in the arterial wall by Virchow et al. [2] in 1856 and the novel article published by Ross et al. [3] in 1999, which highlighted that atherosclerosis is certainly an inflammatory disease, the importance of inflammation and immunity in CVD’s pathophysiology has been a topic of intense basic research.

The nexus between CVD and inflammation is underscored by the role of immune cells, like neutrophils, lymphocytes, macrophages, and monocytes. Hereafter, different scientists took this path of further evaluation of white blood cell (WBC) precursors and their functions in various inflammatory and non-inflammatory disorders. Thus, WBC count and its subtypes, specifically circulating leukocyte-based indices, have emerged as new potential cardiac proinflammatory predictive or prognostic biomarkers.

Total blood count (neutrophil (N), lymphocyte (L), and monocyte (M) counts) and its subtypes have been shown to be an independent risk factor in mortality, especially the neutrophil-to-lymphocyte ratio (NLR) (calculated via dividing neutrophil count by lymphocyte count) [4]. Low-grade inflammation was acknowledged as a common feature of different CVDs. Neutrophils are portrayed as a marker of ongoing inflammation, while lymphocytes are markers of regulatory pathways. The NLR combines two different immune avenues that serve as indicators of inflammation, reflecting, in some way, two aspects of the immune system: inflammation (neutrophil count) and adaptive immunity (lymphocyte count). Although no certain cut-off has been proposed, some studies performed on healthy subjects suggest that the normal NLR values in an adult, non-geriatric, population are between 0.78 and 3.53 [5]. NLR is independently linked to multiple demographic and lifestyle factors (gender, age, race, body mass index, physical activity, alcohol consumption, or smoking history) that contribute to its heterogenic characteristics [6]. NLR was associated with different CVDs, with a higher incidence of adverse outcomes in various clinical situations, and higher mortality in the general population [7], including patients undergoing cardiac surgery [8].

Despite advanced diagnostic tools and novel therapeutic strategies, considering the ongoing evolution of medicine, the pursuit of finding the best preventive strategies is embodied by new biomarkers, indexes, and predictive algorithms that require exhaustive investigation [9]. Therefore, in this literature review, we will display the latest data that has emerged on the prognostic and predictive roles of NLR as a readily available and inexpensive marker that reflects inflammation in the CVDs most frequently encountered in clinical practice and also provide new insight regarding certain questions regarding the utility of this index in clinical practice.

## 2. NLR in Cardiovascular Disease

As discussed, NLR is a widely available hematologic marker of inflammation, oxidative stress, and endothelial damage easily obtained from routine complete blood counts. Meta-analysis studies and other scientific research demonstrated its association with coronary artery disease (CAD), acute coronary syndrome (ACS), stroke, and composite cardiovascular events, proving its application as a predictive/prognostic biomarker in various CVDs [10,11]. The Rotterdam Study, a long-standing, population-based, prospective cohort study, displayed NLR values as indicators for all-cause mortality, cardiovascular mortality, and other mortality, in the elderly population [12]. Interestingly, authors have found, in the Jackson Heart Study (JHS) and validated in the Normative Aging Study (NAS), that in participants with Duffy antigen-negative (ethnic neutropenia via a single-nucleotide polymorphism (rs2814778)) and lower mean neutrophil counts (51.1 per mL^3^), a lower NLR of 1.77 may be a better predictive cut-off value than 2.12 [13]. Hence, when testing NLR as a prognostic marker for health outcomes, it is important to consider population differences (underlying genetic variation, ethnic and racial origin), as the “normal” cut-off values of NLR can be affected by leucocyte number variabilities in these patients.

### 2.1. Heart Failure

HF is one of the most relevant cardiac diseases worldwide, being a clinical syndrome defined not just by a single pathological diagnosis but, instead, by structural and/or functional impairment with different cardiac or non-cardiac etiology. Structural and/or functional abnormality of the heart (e.g., aberrant myocardial collagen deposition) results in elevated intracardiac pressures and/or inadequate cardiac output, resulting in insufficient perfusion. With increased incidence and prevalence of hypertension and chronic ischemia, HF remains a highly burdened disease with elevated health care costs [14,15]. Regardless of the phenotypic diversity [16], HFpEF syndrome reflects a pro-inflammatory state and an oxidative stress pattern that constitutes a major contributor to disease onset and development [17]. Data shows that raised inflammatory markers in all HF stages, including heart failure with reduced ejection fraction (HFrEF) and heart failure with preserved ejection fraction (HFpEF), are associated with many adverse cardiac events. Although there are biomarkers, such as pentraxin-3 and receptors for advanced glycation end products that are specific for HFpEF, they are not available in daily clinical practice [18].

Almost a decade ago, researchers demonstrated that raised-neutrophil lifespan is linked to plasma levels of C-reactive protein (CRP), alkaline phosphatase, and the New York Heart Association (NYHA) class, and could represent a novel measurement of tissue and endothelial dysfunction [19]. Additionally, the Irbesartan in Heart Failure with Preserved Ejection Fraction (I-PRESERVE) trial taught us that higher neutrophils are independent risk factors for poor outcomes in HFpEF individuals [20]. The recent US National Health and Nutrition Examination Survey (NHANES) study, found that elevated neutrophil percentage-to-albumin ratio (NPAR) and NLR, but not platelet-to-lymphocyte ratio (PLR) were independently associated with increased all-cause mortality among adult individuals with HF [21]. Moreover, after investigating 1221 HF patients, the authors noted that a new indicator composed of CRP and NLR (C-NLR) was a reliable predictor for the risk of all-cause mortality in HF patients with different ejection fractions [22]. Bao et al. noted that, in almost 170 patients with (HFpEF), retrospectively investigated NLR coupled with gene signatures (S100A8/A9/A12 and PADI4) was positively associated with hs-CRP, NT-proBNP, and mitral E/e’ [23].

In one study, the authors investigated the inflammatory and nutrition status in heart failure patients with a 1-year and 6-month follow-up. They investigated the predictive value of NLR in major adverse cardiovascular events (MACEs) and its prognostic value by measuring thyroid function via free triiodothyronine (FT3) and the geriatric nutritional risk index (GNRI). It seems that the combination of high NLR and low values of GNRI and FT3 were associated with MACEs [24]. The BIOlogy Study to TAilored Treatment in Chronic Heart Failure (BIOSTAT-CHF), which divided HF patients into two groups: 523 [LVEF] < 40% and 662 LVEF ≥ 40%, noted that elevated NLR was significantly correlated with worse outcome, inflammation-related biomarkers, and cardiac biomarkers such as NT-proBNP [25]. This multicenter trial study noted that a combination of NLR and PLR is a better predictor for cardiac death in subjects with decompensated HF [26]. A recent meta-analysis also displayed the pivotal role of inflammation in CVD, and the role of NLR as a predictive biomarker in short- and long-term mortality in decompensated HF, proving its utility as a tool for risk stratification of these patients [27].

Interestingly, in patients with acute myocardial infarction (AMI) (386 with NSTEMI and 604 with STEMI), NLR independently predicted in-hospital acute HF but the 2 groups did not significantly differ in the occurrence of HF within twelve months of discharge [28]. Regarding acute-HF, the Pre-RELAX-AHF (phase 2b study of recombinant human relaxin-2, serelaxin), RELAX-AHF (phase 3 study of serelaxin), and BLAST-AHF (phase 2b study of the biased ligand of the angiotensin 2 type 1 receptor, TRV027) studies showed that NLR was an independent predictor of 30-day all-cause mortality, 60-day HF/renal failure rehospitalizations or CV death, 180-day CV death and 180-day all-cause mortality, surpassing traditional markers, like natriuretic peptides [29].

In 180 subjects with severe left ventricular ejection fraction (LVEF) dysfunction ≤ 35%, with ischemic or nonischemic HF, and implantable cardioverter-defibrillator (ICD) therapy for primary prevention, NLR (cut-off value 2.69 ng/mL, *p* < 0.01) independently predicted one-year cardiac mortality [30]. In advanced heart failure patients with heart transplantation or mechanical circulatory-assist device recommendations, elevated NLR was associated with increased mortality/heart transplantation risk [31]. Interestingly, one specific study showed that despite the association between NLR, derived neutrophil-to-lymphocyte ratio (dLNR = neutrophils/(leukocytes-neutrophils)), monocyte/granulocyte-to-lymphocyte ratio (MGLR = (white cell count-lymphocyte count) to lymphocyte count), and platelet to lymphocyte ratio (PLR), and short-term mortality, it failed to independently predict the prognosis of HFrEF patients [32]. Moreover, NLR (best cut-off value was 2.41) was a predictor for in-hospital mortality and an independent prognostic factor for one-year mortality in subjects with severe HF and with heart transplantation (HTX) [33].

These results briefly summarise the utility of the NLR index as a predictive or mortality risk assessment biomarker in HF patients, used alone or in combination with other markers.

### 2.2. Acute Coronary Heart Disease

ACSs are characterized by a sudden reduction in blood supply to the heart and include ST-segment elevation myocardial infarction (STEMI), non-STEMI (NSTEMI), and unstable angina. Annually, more than 7 million people are newly diagnosed with ACS, remaining the leading cause of death worldwide [34,35].

In myocardial infarction, because of damaged vascular perfusion and reperfusion, ACS alters the myocardium with triggered local and systematic inflammation that helps with cardiac remodeling [36]. Neutrophils and lymphocytes play a particularly important role in the ACS cascade. Many studies have researched NLR and demonstrated its predictive value for adverse outcomes and mortality in myocardial infarction (MI) conditions, correlated with higher NLR levels. The term “high NLR” was defined by some as a value > 6.69 [37,38]. Arbel et al. [38] investigated the association between NLR and 30-day and 5-year all-cause mortality in patients who underwent primary coronary angioplasty (PCI) for STEMI. Higher NLR (≥6.5%) was independently associated with lower ejection fraction, fewer hospital complications, and higher mortality rates up to five years. A recent systematic review and meta-analysis that included over 20,000 ACS patients undergoing PCI revealed that higher NLR was associated with higher numbers of involved coronaries, with a 3.4 times increase in long-term death and with a higher likelihood of long-term MACEs following the PCI procedure [39]. Complementarily, a recent meta-analysis of ninety studies, including 45,990 patients, demonstrated that NLR was associated with ACS and 30-day risk of MACEs. In STEMI, NLR values differed between subjects withMACEs and without MACEs (6.99 ± 5.27 vs. 4.99 ± 4.12) [40]. Interestingly, one cross-sectional study noted that a value of NLR ≥ 6.42 is a reliable marker in chronic total occlusion in patients with STEMI, and pointed out that smoking was correlated with a seven-fold increase in chronic total occlusion [41].

Oncel et al. [42] showed a proportional increase in NLR, which correlated with the Global Registry of Acute Coronary Events (GRACE) risk score and with MACEs independent of GRACE risk score in patients with STEMI undergoing primary PCI. Neutrophilia can aggravate cardiac ischemia by neutrophil-mediated microvascular plugging and thus enlargement of the infarcted area. In the same registry, others noted that NLR is more accurate than aspartate aminotransferase (AST), LDH, and troponin I (TnI) in predicting cardiac death among NSTEMI vs. STEMI patients. ACS severity is also correlated with the GRACE risk score. The optimal cut-off value of NLR in the NSTEMI group was 5.509 [43]. After adjusting for sex, age, and hypertension as confounders, NLR correlated with the angiographic risk stratification SYNTAX score (a marker of coronary artery disease complexity) in subjects with non-ST-segment elevation acute coronary syndrome (NSTE-ACS). However, after adjustment of the thrombolysis in myocardial infarction (TIMI) score, it seems that the TIMI risk score might be a better predictor of the SYNTAX score vs. NLR [44].

A combination index of NLR and PLT may have a better ability to predict the risk of in-hospital mortality and improve short-term prognosis in ACS patients [45]. Worth mentioning is that even if ACS subjects receive dual antiplatelet therapy and have raised NLR values, they do not achieve enough platelet inhibition, which contributes to thrombosis and enhances the probability of recurring ischemic events [46]. Additionally, as a cost-effective indicator of inflammation, an NLR higher than 3.39 at admission independently predicted all cause-mortality in left main and/or three-vessel disease in patients with AMI [47]. Akpek et al. [48] demonstrated that preprocedural NLR is an independent predictor of no-reflow in patients with STEMI. These results are also sustained by Pinheiro et al. [49] who also noted that significant NLR values are an independent predictor of no-reflow, distal embolization (*p* < 0.001), and procedural complications in STEMI subjects who underwent primary PCI. Earlier this year, a comprehensive meta-analysis highlighted the predictive potential of WBC count, neutrophil count, PLT, hemoglobin, blood glucose, TC, creatinine, d-dimer, and fibrinogen in predicting the risk of the no-reflow phenomenon in STEMI patients after PCI [50]. In contrast, Zhang et al. [51], in the same category of subjects, discovered that even if there is an association, there is no significant difference in the predictive value of NLR, mean platelet volume (MPV), and platelet distribution width (PDW) or their combination.

Given that most of the studies focused on older patients, regarding the coronary no-reflow and death outcomes, elderly STEMI subjects have an initial pro-inflammatory profile more abundant than young subjects, implying that they should have a distinctive therapeutic approach. The values of NLR, leukocytes, neutrophils, fibrinogen, and C reactive protein/albumin ratio (CAR) were associated with no-reflow in elderly patients, while in young patients, only the BNP values were related to no-reflow [52]. In the same line, a cohort study of young individuals with juvenile myocardial infarction at the initial stage and after 3 and 12 months underlines that NLR alone did not exhibit prognostic significance in predicting future mortality. However, alongside other factors (plasma markers of platelet and neutrophil activation, oxidative stress, elastase, and protein oxidation) NLR seems to be a valuable indicator [53]. Upon investigating the combination value as prognostic markers via controlling nutritional status (CONUT) score and NLR, authors showed that a high NLR (>6.07) and CONUT score (>3.5) had worse prognoses in ACS patients. These results indicate that nutritional status and inflammation in CVD are major contributors to disease clinical outcomes [54]. Additionally, a systematic review and meta-analysis of the literature, which included over 16,000 patients, noted that high NLR on-admission was associated with higher overall mortality both in STEMI (*p* < 0.00001) and in patients with NSTEMI (*p* < 0.00001), which appeared to affect clinically important outcomes, including MACE in-hospital and long-term mortality [55]. Similar results were observed in a smaller study of 1550 acute myocardial infarction (AMI) elderly patients aged over 60 years. High NLR (>6.69) was statistically significantly (*p* < 0.05) associated with AMI and with the risk of in-hospital mortality and could represent an independent predictor of poor short-term prognosis [56].

In post-acute myocardial infarction patients, NLR is a predictor of myocardial damage and cardiac dysfunction, linked to a raised risk of mortality and later complications, requiring further confirmation by large randomized clinical trials [57]. Raised CK-mB values, which reflect direct myocardial damage, also correlated with higher NLR levels, ALT, and creatinine, which further emphasizes that systemic inflammation affects other organs in ACS conditions [58]. Upon investigating both novel indexes represented by the systemic immune-inflammatory index (SII) and the derived NLR that reflect the host inflammatory and immune status, in ACS patients undergoing PCI, it was noted that a higher SII or dNLR value was associated with a higher risk of MACEs (all *p* < 0.001) [59]. Interestingly, body mass index (BMI) × albumin (Alb)/NLR defines the decreased advanced lung cancer inflammation index (ALI), which is an independent prognostic risk factor for overall survival in gastroenterological cancers. The authors found that the ALI score is an independent prognostic risk factor for patients with acute coronary syndrome undergoing PCI [60]. Complementarily, one prospective cohort study involving 200 patients with STEMI who underwent pPCI concluded that NLR and SII help predict the risk of no-reflow after pPCI. However, none of them are predictors of the preprocedural TIMI flow grade and the SYNTAX score [61]. NLR > 3.5 was associated with worse one-year survival post-PCI in patients with ACS (*p* < 0.004) being a well-recognized surrogate marker of inflammation [62]. Also, after a 12-week prespecified cardiac rehabilitation regime in patients with unstable ischemic heart disease who underwent successful PCI, inflammatory markers such as NLR, PLR, or hs-CRP were significantly lowered [63].

Therefore, it seems that a deeper exploration of the role of NLR in early-onset ACS and its inclusion in cardiovascular risk scores may improve risk stratification for negative cardiac events in hospital or at discharge [64].

### 2.3. Atherosclerosis and Chronic Coronary Heart Disease

As is known, the atherosclerosis (ATS) process is composed of a myriad of inflammatory pathways, and neutrophils and lymphocyte progenitors play a particularly vital role in CVD. The formation of AST plaques despite the arterial territory interest, leads to chronic or acute atherosclerotic cardiovascular disease (ASCVD), including systemic ATS, carotid ATS, peripheric arterial disease, or chronic CAD.

The Copenhagen General Population Study, which included over 100,000 subjects, researched the correlation between neutrophil counts and risk of 9 cardiovascular endpoints via observational and genetic approaches. They concluded that high neutrophil counts were associated with high risks of all outcomes in ASCVD, with similar results being observed in men and women [65]. One retrospective study with 4000 patients noted that the Chinese visceral adiposity index (VAI) and NLR were independent risk factors for carotid atherosclerosis and showed positive associations with the 10-year ASCVD risk score (all *p* < 0.001) [66]. Wang et al. [67] also delineated the utility of NLR and plasma lipoprotein(a) (Lp(a)) in predicting unstable coronary artery plaques in 1618 patients with ASCVD, detected on coronary computed tomography angiography (CTA). On the same spectrum, other researchers identified NLR as a novel and meaningful biomarker for predicting the presence of vulnerable carotid plaque assessed by carotid ultrasonography and carotid plaque vulnerability [68], suggesting its role in identifying the risk of harboring carotid plaques [69,70].

CAD is ranked as the third cause of CVD death, accounting for over 17 million deaths globally [71]. As some patients remain asymptomatic, it is imperative for early detection of CAD and identification of risk patients. Bagyura et al. [72] brought attention to the correlation between subclinical, chronic, and systemic inflammation and subclinical CAD in central obesity. Data from 280 asymptomatic participants indicated that NLR is associated with coronary artery calcium score (CACS) > 100 in the 3rd VAI tertile. Very recently, in 1161 Thai physicians who participated in the “Save Doctors’ Heart” project significantly higher levels of neutrophils, NLR, and WBC, and lower platelets were found; NLR > 1.87 and PLR > 161.66, age > 50 years, and coronary artery calcium (CAC) score > 1 were found to be independent factors predicting predict coronary artery disease (CAD) [73]. Authors have investigated the role of NLR, C-reactive protein–albumin ratio (CAR), and platelet–lymphocyte ratio (PLR) in the prognosis of patients with co-presentation of CAD complicated with COVID-19. While a higher CAR trend corresponded to a higher risk of cardiovascular and respiratory failure death, the variations of NLR and PLR did not affect these risks [74]. NLR predicted long-term clinical outcomes of stable CAD patients who underwent elective percutaneous coronary intervention (PCI) and had peri-procedural low high-sensitivity C-reactive protein (hs-CRP) levels (<2.0 mg/L) [75]. Moreover, the calculated cut-offs for NLR > 3.1 and PLR > 204.4 were associated with increased risk of 30-day MACEs. When combined with hs-cTnT or NT-proBNP, NLR improved risk prediction in CAD individuals undergoing non-cardiac surgery [76].

In the National Health and Nutrition Examination Survey-III, the NLR independently predicted cardiovascular mortality in the general population with asymptomatic coronary heart disease (CHD), despite the traditional Framingham risk factors. It improved the intermediary risk score, which emphasizes the beneficial role of NLR in improving current risk scores for CVD [77]. Years after, a population-based study composed of 9409 individuals investigated the predictive value of NLR and remnant cholesterol (Remnant-C) concerning cardiovascular events and all-cause mortality by analyzing data from the National Health and Nutrition Examination Survey (NHANES). NLR exhibited a positive correlation with Remnant-C (*p* < 0.001), and both marked their potential predictive markers for cardiovascular events in the general population [78]. Recently, upon investigating the EMPA-HEART CardioLink-6 trial regarding therapy with sodium–glucose transport protein 2 inhibitor in six-month regression in left ventricular mass in CAD and diabetic patients, the authors pointed out the treatment has beneficial effects independent of baseline NLR [79]. NLR may not only be a good predictor for ATS, but it also exhibits prognostic value regarding future cardiovascular events.

### 2.4. Hypertension

Arterial hypertension (HTN) is portrayed by an inflammatory process that incorporates the transmigration and collection of both innate and adaptive immune cells into the interstitium of affected organs [80]. A link between white blood cell count and blood pressure levels has been established over the years [81,82], with NETosis contributing to HTN via NET-mediated endothelial cell (EC) dysfunction [83]. A cross-sectional study displayed a correlation between higher NLR levels (NLR > 2.7) and blood pressure (BP) variability, suggesting its role as a marker to indicate an increased risk of HTN-related adverse cardiovascular events [84]. A non-dipping blood pressure pattern is associated with higher cardiovascular mortality. As chronic inflammation plays an essential role in the pathophysiology of both HTN and CAD, this cross-sectional retrospective analysis demonstrated that NLR with MLR and PLR work as biomarkers to predict a non-dipping pattern in hypertensive patients with stable CAD [85]. One meta-analysis reviewed the evidence for differences in NLR between hypertensive and non-hypertensive individuals and among subjects with non-dipper hypertension and dipper hypertension profiles. It revealed a significant increase in NLR levels for the hypertensive group (*p* < 0.0001), especially in the non-dipper group [86]. In this matter, Sunbul et al. [87] demonstrated that patients with non-dipper hypertension had significantly higher NLR and PLR compared to those with dipper hypertension. In hypertensive patients, the NLR, BNP, and CRP levels were higher in left eccentric and concentric hypertrophy (left ventricular hypertrophy (LVH)), compared to the non-LVH group [88]. HTN patients had higher NLR, CRP, and brain natriuretic peptide (BNP) levels, which correlated with eccentric and concentric ventricular hypertrophy (LVH) [88]. Interestingly, HTN in elderly subjects with a higher quartile of NLR and red cell distribution width (RDW) was linked to higher all-cause mortality at 90 days after admission [89]. Also, HTN patients had higher values of NLR associated with diastolic dysfunction, which raises the utility of NLR as a marker for prognostic stratification in diastolic dysfunction [90].

Pulmonary hypertension (PH), which can appear in approximately 50% of patients, is usually observed in patients with severe aortic stenosis (AS), being a predictor of adverse outcomes after transcatheter aortic valve replacement (TAVR). Recently, authors demonstrated that higher baseline NLR is associated with increased 3-month all-cause mortality in patients with periprocedural PH and valvular aortic disease (AS and aortic regurgitation) who underwent TAVR [91]. In patients with PH and left ventricular diastolic dysfunction (LVDD), NLR partially mediated the effect of adverse uric acid outcomes, such as the risk of all-cause mortality, HF hospitalization, and cardiac death [92].

Interestingly, in 119 pediatric subjects with primary hypertension (Ph), NLR and PLR were demonstrated to be markers of arterial damage/stiffness. Specifically, NLR positively linked (*p* < 0.05) with diastolic, systolic, and mean blood pressure in ABPM and with common carotid artery PwVbeta [m/s] [93]. These discoveries highlight the reliability of inflammatory markers, such as NLR, not only in the adult population with HTN but also in younger patients, which opens new paths for further studies.

### 2.5. Cardiac Arrhythmias—Atrial Fibrillation

According to the task force for the diagnosis and management of atrial fibrillation (AF) of the European Society of Cardiology (ESC), developed with the special contribution of the European Heart Rhythm Association (EHRA), AF is one of the most common cardiac arrhythmias encountered in clinical practice, with increased incidence depending on age (more than 10% of people > 80 years of age) [94], increased all-cause mortality of the population almost by 1.5–3.5-fold [95], and great disability [96]. An inflammatory pattern causes and accelerates the electrical and structural remodeling of the atrial cardiomyocytes by the release of pro-inflammatory cells, with subsequent fibroblast activation and fibrosis damage. This promotes the development, maintenance, and the outcome and prognosis of AF. Further inflammation forms a vicious spiral, so-called “AF begets AF” [97]. Complementarily, analyses from the ENGAGE AF- TIMI 48 trial revealed that baseline NLR was statistically associated with MACEs, major bleeding events, cardiovascular death, stroke/systemic embolism, and all-cause mortality [98]. While investigating the Analysis of the Multi-Parameter Intelligent Monitoring in Intensive Care (MIMIC-IV) database, a large cohort of 4562 critically ill patients with AF, the authors found that NLR, PLR, and SII, were linearly associated with 30-day and 365-day risk of mortality [99]. The Atrial Fibrillation and Perioperative Inflammation (FIBRILLAMMED) study marked the inflammatory response in off-pump coronary artery bypass grafting (OPCABG) settings in 151 patients; a value of aa-NLR ≥ 1.32, aa-PLR ≥ 52.64, and aa-SII ≥ 344.38 predicted new-onset atrial fibrillation (NOAF) [100].

In a high rate of cases of patients undergoing catheter ablation (CA) for AF who experience recurrence of arrhythmia, a panel of pre-ablation serum biomarker(s) may improve patient selection before CA [101]. Patients with late nonvalvular atrial fibrillation (NVAF) recurrence after radiofrequency ablation (RFA) had significantly statistically higher NLR, high-sensitivity C-reactive protein (hs-CRP), left ventricular end-systolic dimension (LVESD), left ventricular end-diastolic dimension (LVEDD), and left atrial diameter (LAD) compared to the non-recurrent group [102]. Others noted that a combination of preoperative echocardiographic left atrial diameter LAD, NLR, and hs-CRP (with cut-off values of 44.5 mm, 2.33, and 2.025 ng/L) may predict late nonvalvular atrial fibrillation after radiofrequency ablation [102].

In addition, NRL has been reported as a perioperative prediction biomarker regarding the incidence of postoperative atrial fibrillation (POAF) after cardiac surgery. Following cardiac surgery, the most common arrhythmia complication is postoperative atrial fibrillation (POAF) classified as acute POAF (between the 1st and the 4th postoperative day) or subacute (sPOAF) (between the 5th and the 30th). In this case, acute POAF and NLR at baseline were noted to be independent predictive factors of sPOAF after heart surgery [103]. A recent meta-analysis that incorporated 12 studies and 9262 participants noted that preoperative NLR was not a significant predictor of POAF after correction for covariates that contributed to heterogeneity and changed values [104]. Similar results by Jacob et al. [105] described, in a large cohort study of 277 (42%) preoperative sinus rhythm patients with elective cardiac surgery, that NLR and other white blood cell precursors did not associate with secondary POAF. After heart transplantation, in subjects with end-stage heart failure, preoperative neutrophil-to-white cell ratio (NWR), but not NLR, was linked with the risk of documented paroxysmal AF in the first 2 months [106].

Early detection and treatment of cardiac arrhythmias, such as AF, are crucial in clinical practice, given their associations with cardiomyopathies, left ventricular dysfunction over time, and poor clinical outcomes [107].

### 2.6. Valvular Heart Disease

According to recent new guidelines regarding valvular heart disease (VHD), the incidence of degenerative etiology has increased in industrialized countries, while, among the imagistic options for diagnosis, such as non-invasive evaluation using three-dimensional (3D) echocardiography, biomarkers also play a more and more central role. Current guidelines bring to attention the heart team and heart valve centers (centers performing heart valve procedures with institutional cardiology and cardiac surgery departments with 24 h/7-day services) as the main evaluation step for better diagnosis and VHD treatment [108,109].

In mechanical stress conditions, which enhance radical hemodynamics, or in primary congenital valves that are prone to damage, valve interstitial cells (VICs), the conductors of valve homeostasis, are stimulated by a pro-inflammatory cascade and then transformed from quiescent VICs to activated VICs, which triggers calcification and sclerosis. Among the inflammation pathways and metabolic regulation of osteogenesis, attention is required regarding the expression of metalloproteinases (MMPs) that participate in the remodeling of tissues and leukocyte density, which are correlated with the expression of tumor necrosis factor-α and the hemodynamic progression rate [110].

Calcific aortic valve disease (CAVD) is dominated by a similar mechanism to coronary/carotid artery atherosclerosis via chronic inflammation regulation. In these lines, NLR utility as a biomarker was demonstrated in mitral annular calcification [111,112] and in all grades of degenerative aortic stenosis [113]. NLR and CRP were investigated in 123 CAVD subjects with bicuspid aortic valve (BAV) and others with tricuspid aortic valve (TAV) vs. 108 healthy individuals. NLR and CRP showed their predictive potential in both valvular diseases; NLR, however, will not extend the cardiopulmonary bypass time (CPB), but it will prolong the operation time and the postoperative mechanical ventilation time. In over 3000 patients with heart valve surgery, a value of CRP levels > 5 mg/L predicted postoperative heart failure, while NLR > 3.5 had a higher incidence of death within 30 days after surgery (*p* < 0.001) [114]. Additionally, NLR, PLR, a history of arterial hypertension, and smoking history were independently associated with the presence of CAVD [115].

After heart surgical procedures such as TAVR, a systemic inflammatory response can occur, which is reported to be independently correlated with higher mortality [116]. As previously mentioned, higher baseline NLR was independently correlated with adverse outcomes, including all-cause mortality and HF rehospitalization in TAVR patients. In procedural transcatheter aortic valve implantation (TAVI), an improvement in the inflammatory profile was observed after 6 months (NLR 2.94) [117]. Upon investigating the short- and long-term mortality in patients with AS undergoing surgical treatment with TAVR, the authors found that NLR is an independent predictor of short-term and long-term mortality (3-years), especially for those with NLR ≥ 3 [118]. Furthermore, in TAVR subjects, the association between NLR and PLR with baseline characteristics was linked with the occurrence of 30-day adverse outcomes, similar to the Society of Thoracic Surgeons Predicted Risk of Mortality (STS-PROM) score [119].

When studying the prognostic role of NRL in CVD, we have noticed that most relevant studies evaluated the risk of all-cause mortality and short- and/or long-term MACEs, mainly in chronic cardiac disease, while studies that investigated acute CVDs had, among the primary outcomes, cardiac mortality. Although, the prognostic value of NLR is investigated over a 1-, 3-, or 6-month follow-up, some studies also researched 1 year or 3 years after its measurement (Table 1).

## 3. Current Trials That Involve NLR in Cardiovascular Disease

There are currently a few government-approved clinical trials that are investigating the role of NLR in different CVD diseases and exhibiting their results in known scientific journals. For example, in 1026 patients registered in the Prospective Multicenter Observational Study of Patients with Heart Failure with Preserved Ejection Fraction (UMIN000021831), high-admission NLR (>4.5) and PLR (>193), were independently associated with cardiac death, and a combination of both values was the strongest predictor [26]. One trial demonstrated the association of NLR as a predictor of CAD and carotid intima-media thickness in dialysis patients (NCT05472805) [120]. An older trial (NCT01663194) sought to show the association between pre-procedural NLR within the hospital and long-term outcomes among STEMI patients undergoing PCI [121]. Also, the NCT02828137 trial noted that NLR is associated with coronary microcirculation assessed by the index of microcirculatory resistance (IMR) in STEMI patients [122]. The Placement of Aortic Transcatheter Valves (PARTNER) I, II, and S3 trials and registries (NCT00530894) for severe aortic stenosis receiving TAVR or SAVR showed that raised admission NLR was associated with an increased risk of increased subsequent mortality and rehospitalization at 3 years, irrespective of treatment option [123]. The perspective of novel research about the utility of NLR in CVD is desired; hopefully, scientists will soon develop trials that will help the community with new data that can be used for clinical practice.

## 4. Discussion and Conclusions

Every year, the number of individuals admitted to hospitals with CVD increases; thus, early diagnosis is crucial to prevent the impairment of heart and vessel function. As can be seen, not only does a vast amount of preclinical and observational evidence highlight the pivotal role of inflammation in various CVDs, but it also provides the necessary basis for developing novel nomograms containing predictive and prognostic markers, such as NLR.

As Bhat et al. [10] and 3 years later, Afari et al. [124], previously discussed this topic, the purpose of this review is not only to bring forth the recent scientific discoveries about the role of NLR in CVDs but also to provide new insight into trying to answer certain questions regarding the utility of this index in clinical practice.

### 4.1. Are There Other Mechanisms Involved in NLR in Addition to Inflammation?

In addition to inflammation and reduced immune function in CVDs, neutrophils and lymphocytes are jointly regulated through complex mechanisms. Accordingly, researchers suggest a more specific mechanism that involves the stimulated release of immature and mature neutrophils that can induce suppression of T-cell proliferation [125,126]. These cells are the granulocytic myeloid-derived suppressor cells (gMDSCs) released from the bone marrow secondary to chronic/acute disease impairment (e.g., acute myocardial infarction). Morphologically similar to granulocytes and monocytes, MDSCs have strong immunosuppressive activity [127,128]. The immunoregulatory gMDSCs increase up to 10% of the peripheral blood leukocytes by concomitantly suppressing the lymphocyte response; thus, it can be stipulated that NLR is a measure of the phenotypic activity of gMDSCs [7,13]. Further research, however, is required to elucidate this supposition.

### 4.2. Is It the Leucocyte Number or the Differential L and N Numbers That Predict Prognosis? Are the NLR Ranges of Those with and without Disease Different, or Are They the Same for the Different CVDs?

To answer these questions, we further explored the results from previous studies. Firstly, Bhat et al. [10], in their review, pointed out that greater predictive ability for acute events in CAD patients is provided by high neutrophil or low lymphocyte counts, and that the greatest risk prediction is provided by NLR. Complementarily, Horne et al. [4] also raised the question about which leukocyte subtypes carry cardiovascular risk. In this matter, in patients without acute MI but with CAD, the greater predictive ability was provided by a high NLR, followed by high neutrophil or low lymphocyte counts. More specifically, they noted that a relatively high neutrophil count and a relatively low lymphocyte count account for WBC count risk (Q4 ratios NLR > 4.7 with elevating risk three-fold). On this topic, some older epidemiological studies showed that the monocyte values correlated with increased risk [129], while others showed that neutrophil counts are reliable in risk assessment [130].

The values of NLR vary depending on the disease status of the patients and on the time of determination. For example, Song et al. [7] found that NLR was linked with CVDs over long intervals of follow-up, such as 12–49 and 50–93 months, for heart disease, and in subjects without these conditions at baseline. Patients with a baseline history of related heart disease had NLR values of 1.06 (0.95–1.25), versus subjects with other conditions chronic lower respiratory diseases 1.24, (1.04–1.47), or kidney diseases 1.62, (1.21–2.17), all diseases being associated with higher mortality compared to without those conditions at baseline. Additionally, others reported that NLR levels were positively associated with elevated mortality from CVDs up to 8 years after baseline measurement [12]. Angkananard et al. [11,131] observed that the NLR cut-off points ranged from 1.80 to 2.60, and reported mean differences in NLR between CAD and non-CAD patients.

It seems that those with noncardiac chest pain reported the lowest admission NLR (3 ± 1.6), while unstable angina, NSTEMI, and STEMI, registered NLR higher values of (3.6 ± 2.9), (4.8 ± 3.7), and (6.9 ± 5.7), respectively. Elevated preoperative NLR (>3.36) has been associated with worse outcomes after CABG [10]. Consistent with these results, Shao et al. [132] performed a comprehensive meta-analysis that included almost 2800 subjects and noted that incident AF for baseline NLR level was 1.25, and after CABG, RFCA, and cardioversion, NLR was 1.518, with significant heterogeneity across studies.

These results highlight that NLR value depends on the presence or absence of certain diseases, disease severity, and onset (acute/chronic), before or after a procedure, the time of detection, and the follow-up detection. Different outcomes may also be due to different study populations and NLR levels among various researchers. Also, NLR assays performed on other occasions may be another possible reason for the contradictory results among the different studies.

Considering the robust data investigated, a better overview of these differences and variety of NLR values in CVDs can be seen in Table 2.

### 4.3. What Are the Limitations in Establishing a Universal Cut-Off Value for NLR?

As observed, NLR is a widely available, inexpensive marker that is simply calculated from the routinely performed white blood cell count early on, upon initial presentation to any elective evaluation or the emergency department. However, some points were raised in the first reviews written by experts, such as the need to establish clinically relevant and consistent cutoffs for high and low NLR.

Recent data provides new insight regarding this issue. Firstly, a general cut-off is difficult to assess because, as mentioned above, NLR is characterized by heterogeneity. It is important to mention that physiological lymphocyte production decreases with age, and therefore NLR increases; thus, we assume that in disease or non-disease conditions, the value of NLR is different according to age groups (lower in the younger and higher in the elderly population). Also, many other factors, such as gender, race, body mass index, and physical activity, contribute to NLR heterogeneity. For example, in a subgroup analysis performed by Angkananard et al. [11], their systematic review noted that higher values of NLR were found in Asians (2.87) than Caucasians (1.79), and greater in patients ≤ 65 years (2.07) than in patients > 65 years. Because neutrophil and lymphocyte production are influenced by lifestyle factors, such as smoking history, or association with other non-cardiac inflammatory diseases (e.g., sepsis, cancer), many studies excluded subjects that have these associations.

Additionally, every inflammatory response is somewhat different, depending on the underlying disease and its status, whether acute or chronic (values of NLR between 4 to 6 or higher in ACS vs. 2–4 in other chronic CVDs). For example, in an acute setting, such as STEMI or NSTEMI, the sudden inflammatory condition affects the rheology of neutrophils, with secondary rapidly increased numbers and secondary activation of a gamut of pathways for tissue repair. However, the total number of leukocytes constitutes all the different subgroups; all changes cannot be reflected by a single measurement. Most of the studies mention that STEMI is detected at baseline with neutrophilia, which predicts a larger infarction and a low baseline number of lymphocytes. A higher NLR, in these cases, is attributed, in most studies, to higher neutrophil levels. We could not identify, in the literature, studies that differentiate NLR from neutrophil counts as predictors of events in CVDs. Ultimately, NLR value is most likely secondary to the individualized immune response.

### 4.4. Is NLR Influenced by Anti-Inflammatory Therapies?

To answer this question, we bring to attention the results of the analyses from five contemporary randomized trials (CANTOS, JUPITER, SPIRE-1, SPIRE-2, and CIRT trials) of canakinumab, rosuvastatin, bococizumab, or methotrexate vs. placebo in ATS patients. The results showed that lipid-lowering agents had no significant impact on the NLR, while anti-inflammatory therapy with canakinumab (human monoclonal antibody targeted at interleukin-1 beta) significantly lowered the NLR [134], which raises another question about other therapies and anti-interleukin molecules and their effects on NLR.

### 4.5. Are There Any Longitudinal Studies?

When we discuss the validation of certain biomarkers for clinical utility, the data obtain from longitudinal studies are better for establishing the correct sequence of events, identifying changes over time, and providing insight into cause-and-effect relationships.

We have identified a few longitudinal studies with long-term follow-up assays of NLR. Among them, we mention The Rotterdam study, which conducted periodic evaluations every 3–4 years from 1990 until 2008 [12], and the NHANES study, with a median follow-up of 59.3 months [21]. Additionally, the CANTOS trial, which followed primary and secondary outcomes at 48 months, demonstrated that NLR levels were stable over time, raising the potential for their use as a clinical biomarker. Further, longitudinal studies are required to answer some of the questions regarding NLR.

Finally, despite the burgeoning interest, there remains a paucity of data exploring these parameters and many other questions to be answered. Evidence shows that even if NLR’s standalone predictive performance for a specific condition can be limited, given its heterogeneity, finding a specific cut-off value for each disease, and when used in combination with other inflammatory markers, its utility may be proven in clinical practice [134]. Complementarily, its addition to risk scores may raise its predictive and prognostic performance and therefore improve short- and long-term CVD outcomes.

## Figures and Tables

**Table 1:** Relevant studies involving NLR in CVDs.

Study Type	Condition	Patient Number	NLR Cutoff Point	Cardiac Mortality versus Overall Mortality Prediction?	Salient Findings	Ref.
MPO study	HF	1622	3.22	all-cause mortality and/or HF hos pitalization	NLR was significantly associated with the primary outcome (*p* < 0.001); despite LVEF, NLR was significantly correlated with biomarkers related to inflammation as well as NT-pro-BNP.	[25]
PMO Study	D-HF	1026	≤4.5	cardiac and all-cause death, respectively	High NLR and PLR values were independently linked with cardiac death, and a combination of both values was the strongest predictor (*p* = 0.0008)	[26]
Retrospective cohort	Acute HF	549	4.78	In hospital all-causes mortality	NLR significantly associated with the primary outcome (OR 1.156, 95% CI 1.001–1.334, *p* = 0.048)	[31]
Prospective single-center registry	STEMI	538	6.5	In hospital clinical cardiac mortality	High NLR (NLR ≥ 6.5%) was independently associated with increased 30-day and 5-year mortality rates, independently associated with lower EF (49 ± 8 vs. 46 ± 8; *p* < 0.001) and fewer hospital complications.	[38]
Retrospective observational study	STEMI	101	-	in-hospital cardiac mortality	Occurrence of reinfarction or new-onset heart failure was significantly related to NLR at admission (*p* < 0.001). NLR and GRACE risk score showed a significant positive correlation (r = 0.803, *p* < 0.001).	[42]
Prospective cohort	ACS	1553	2.29	MACEs, which included all-cause mortality and rehospitalization	Higher SII or dNLR value was associated with a higher risk of MACEs (all *p* < 0.001)	[59]
Retrospective cohort study	CAD	1951	1.9	Cardiac mortality	Increasing NLR as a continuous variable was associated with the incidence of adverse cardiovascular events (HR 1.85 per log 1 NLR increase, 95% CI 1.19–2.88, *p* = 0.007).	[75]
Post hoc analysis	CHD	7363	2.68	Cardiac mortality	NLR can independently predict CHD mortality; it reclassifies intermediate risk category of FRS	[77]
Retrospectively single center study	PH	128	-	All-cause mortality	Association between higher NLR, increased risk of periprocedural PH, and increased 3-month all-cause mortality (16.1% vs. 3.1% in lower NLR group, *p* = 0.021)	[91]
cohort study	AS	-	3	All-cause mortality	NLR ≥ 3, had a significantly higher short-term (9.40% vs. 0, *p* = 0.0006), 6-month (19.54% vs. 0.95%, *p* < 0.0001), and 3-year mortality (27.35% vs. 3.78%, *p* < 0.0001)	[118]

multicenter, prospective, observational study (MPO); major adverse cardiovascular events (MACEs); prospective multicenter observational study (PMO); left ventricular ejection fraction (LVEF), myocardial infarction (MI); neutrophil percentage-to-albumin ratio (NPAR); neutrophil-to-lymphocyte ratio (NLR); platelet-to-lymphocyte ratio (PLR); coronary heart disease (CHD); Framingham risk score (FRS); systemic immune-inflammatory index (SII); postoperative atrial fibrillation (POAF); monocyte-to-lymphocyte ratio (MLR); left ventricular hypertrophy (LVH); nonvalvular atrial fibrillation NVAF); high-sensitivity C-reactive protein (hs-CRP); left ventricular end-systolic dimension (LVESD), left ventricular end-diastolic dimension (LVEDD), and left atrial diameter (LAD); calcific aortic valve disease (CAVD); “-” not specified.

**Table 2:** Mean difference of NLR and neutrophils reported between CVD and non-CVD patients in systematic reviews and meta-analysis studies. acute decompensated heart failure (ADHF); occurrence after coronary artery bypass grafting (CABG); or radiofrequency catheter ablation (RFCA) or cardioversion (CV).

Studies	CVDs vs. Non-CVDs	Neutrophils Count	Cut off Values
2013-Bhat et al. [10]	CAD (2.5–5.19 ± 3.81 vs. 1.96–3.00) NSTEMI (4.8 ± 3.7 STEMI (6.9 ± 5.7) Ventricular arrythmia (3.79 vs. 1.56) ADHF (9.6 (7.6–13.1), 5.1 (4.5–5.8) and 2.8 (2.2–3.8) CABG preoperative (3.0 vs. 2.4) and postoperative (9.2 vs. 7.2)	Ventricular arrythmia (75.79 vs. 58.06%)	-
2016-Shao et al. [132]	Incident AF (1.16–1.35) Post NLR levels (1.076–2.142) AF recurrence after CABG, RFCA and cardioversion (1.108–2.079)	-	Incident AF (1.25) Post AF (1.518)
2018-Angkananard et al. [11]	CAD (2.37–5.66 vs. 1.51–4.30) ACS (2.38–5.58 vs. 1.82–5.10) Stroke (1.40–5.60 vs. 1.40–3.60)	CAD (31–691 vs. 33–352) ACS (38–349 vs. 34–251) Stroke (38–200 vs. 30–140)	CAD (1.80–2.60) ACS (2.19–5.70) Stroke (3.00–3.17)
2020 Liu et al. [104]	POAF (3.4–8.5)	-	POAF (2.6)
2023 Vakhshoori et al. [133]	HF (4.38)	-	HF (1.27–2.46)
2023 Sarejloo et al. [86]	Dipper-HTN (1.80–2.02 ± 1.32), Non-Dipper HTN (1.58–3.10± 0.95) vs. control (1.38–2.13 ± 0.87)	Dipper-HTN (28–269) vs. Non-Dipper HTN (30–266) vs. control (13–132)	-
2024 Shahsanaei et al. [39]	PCI-ACS (2.325–5.025)	-	PCS-ACS (1.021–1.353)

## Data Availability

No new data were created or analyzed in this study. Data sharing is not applicable to this article.

## References

[1] Murray C.J., Aravkin A.Y., Zheng P., Abbafati C., Abbas K.M., Abbasi-Kangevari M., Abd-Allah F., Abdelalim A., Abdollahi M., Abdollahpour I. (2020). Global burden of 87 risk factors in 204 countries and territories, 1990–2019: A systematic analysis for the Global Burden of Disease Study 2019. Lancet.

[2] Virchow R. (1856). Der ateromatose prozess der arterien. Wien. Med. Wochenschr..

[3] Ross R. (1999). Atherosclerosis—An inflammatory disease. N. Engl. J. Med..

[4] Horne B.D., Anderson J.L., John J.M., Weaver A., Bair T.L., Jensen K.R., Renlund D.G., Muhlestein J.B. (2005). Which white blood cell subtypes predict increased cardiovascular risk?. J. Am. Coll. Cardiol..

[5] Forget P., Khalifa C., Defour J.P., Latinne D., Van Pel M.C., De Kock M. (2017). What is the normal value of the neutrophil-to-lymphocyte ratio?. BMC Res. Notes.

[6] Howard R., Scheiner A., Kanetsky P.A., Egan K.M. (2019). Sociodemographic and lifestyle factors associated with the neutrophil-to-lymphocyte ratio. Ann. Epidemiol..

[7] Song M., Graubard B.I., Rabkin C.S., Engels E.A. (2021). Neutrophil-to-lymphocyte ratio and mortality in the United States general population. Sci. Rep..

[8] Silberman S., Abu-Yunis U., Tauber R., Shavit L., Grenader T., Fink D., Bitran D., Merin O. (2018). Neutrophil-Lymphocyte Ratio: Prognostic Impact in Heart Surgery. Early Outcomes and Late Survival. Ann. Thorac. Surg..

[9] Islam M.M., Satici M.O., Eroglu S.E. (2024). Unraveling the clinical significance and prognostic value of the neutrophil-to-lymphocyte ratio, platelet-to-lymphocyte ratio, systemic immune-inflammation index, systemic inflammation response index, and delta neutrophil index: An extensive literature review. Turk. J. Emerg. Med..

[10] Bhat T., Teli S., Rijal J., Bhat H., Raza M., Khoueiry G., Meghani M., Akhtar M., Costantino T. (2013). Neutrophil to lymphocyte ratio and cardiovascular diseases: A review. Expert. Rev. Cardiovasc. Ther..

[11] Angkananard T., Anothaisintawee T., McEvoy M., Attia J., Thakkinstian A. (2018). Neutrophil Lymphocyte Ratio and Cardiovascular Disease Risk: A Systematic Review and Meta-Analysis. Biomed. Res. Int..

[12] Fest J., Ruiter T.R., Groot Koerkamp B., Rizopoulos D., Ikram M.A., van Eijck C.H.J., Stricker B.H. (2019). The neutrophil-to-lymphocyte ratio is associated with mortality in the general population: The Rotterdam Study. Eur. J. Epidemiol..

[13] Kim S., Eliot M., Koestler D.C., Wu W.C., Kelsey K.T. (2018). Association of Neutrophil-to-Lymphocyte Ratio with Mortality and Cardiovascular Disease in the Jackson Heart Study and Modification by the Duffy Antigen Variant. JAMA Cardiol..

[14] McDonagh T.A., Metra M., Adamo M., Gardner R.S., Baumbach A., Böhm M., Burri H., Butler J., Čelutkienė J., Chioncel O. (2021). 2021 ESC Guidelines for the diagnosis and treatment of acute and chronic heart failure: Developed by the Task Force for the diagnosis and treatment of acute and chronic heart failure of the European Society of Cardiology (ESC) with the special contribution of the Heart Failure Association (HFA) of the ESC. Eur. Heart J..

[15] McDonagh T.A., Metra M., Adamo M., Gardner R.S., Baumbach A., Böhm M., Burri H., Butler J., Čelutkienė J., Chioncel O. (2023). 2023 Focused Update of the 2021 ESC Guidelines for the diagnosis and treatment of acute and chronic heart failure: Developed by the task force for the diagnosis and treatment of acute and chronic heart failure of the European Society of Cardiology (ESC) with the special contribution of the Heart Failure Association (HFA) of the ESC. Eur. Heart J..

[16] Borlaug B.A., Sharma K., Shah S.J., Ho J.E. (2023). Heart Failure with Preserved Ejection Fraction: JACC Scientific Statement. J. Am. Coll. Cardiol..

[17] Aimo A., Castiglione V., Borrelli C., Saccaro L.F., Franzini M., Masi S., Emdin M., Giannoni A. (2020). Oxidative stress and inflammation in the evolution of heart failure: From pathophysiology to therapeutic strategies. Eur. J. Prev. Cardiol..

[18] Tromp J., Khan M.A.F., Mentz R.J., O’Connor C.M., Metra M., Dittrich H.C., Ponikowski P., Teerlink J.R., Cotter G., Davison B. (2017). Biomarker Profiles of Acute Heart Failure Patients with a Mid-Range Ejection Fraction. JACC Heart Fail..

[19] Tracchi I., Ghigliotti G., Mura M., Garibaldi S., Spallarossa P., Barisione C., Boasi V., Brunelli M., Corsiglia L., Barsotti A. (2009). Increased neutrophil lifespan in patients with congestive heart failure. Eur. J. Heart Fail..

[20] Komajda M., Carson P.E., Hetzel S., McKelvie R., McMurray J., Ptaszynska A., Zile M.R., Demets D., Massie B.M. (2011). Factors associated with outcome in heart failure with preserved ejection fraction: Findings from the Irbesartan in Heart Failure with Preserved Ejection Fraction Study (I-PRESERVE). Circ. Heart Fail..

[21] Wu C.C., Wu C.H., Lee C.H., Cheng C.I. (2023). Association between neutrophil percentage-to-albumin ratio (NPAR), neutrophil-to-lymphocyte ratio (NLR), platelet-to-lymphocyte ratio (PLR) and long-term mortality in community-dwelling adults with heart failure: Evidence from US NHANES 2005–2016. BMC Cardiovasc. Disord..

[22] Yang F., Zhang L., Huang W., Liu D., Yang Y., Gu W., Shi T., Yang S., Chen L. (2024). Clinical prognostic impact of C-NLR in heart failure patients with different ejection fractions: A retrospective study. BMC Cardiovasc. Disord..

[23] Bai B., Cheng M., Jiang L., Xu J., Chen H., Xu Y. (2021). High Neutrophil to Lymphocyte Ratio and Its Gene Signatures Correlate with Diastolic Dysfunction in Heart Failure with Preserved Ejection Fraction. Front. Cardiovasc. Med..

[24] Liu L., Chen Y., Xie J. (2022). Association of GNRI, NLR, and FT3 with the Clinical Prognosis of Older Patients with Heart Failure. Int. Heart J..

[25] Curran F.M., Bhalraam U., Mohan M., Singh J.S., Anker S.D., Dickstein K., Doney A.S., Filippatos G., George J., Metra M. (2021). Neutrophil-to-lymphocyte ratio and outcomes in patients with new-onset or worsening heart failure with reduced and preserved ejection fraction. ESC Heart Fail..

[26] Tamaki S., Nagai Y., Shutta R., Masuda D., Yamashita S., Seo M., Yamada T., Nakagawa A., Yasumura Y., Nakagawa Y. (2023). Combination of Neutrophil-to-Lymphocyte and Platelet-to-Lymphocyte Ratios as a Novel Predictor of Cardiac Death in Patients with Acute Decompensated Heart Failure with Preserved Left Ventricular Ejection Fraction: A Multicenter Study. J. Am. Heart Assoc..

[27] Ang S.P., Chia J.E., Jaiswal V., Hanif M., Iglesias J. (2024). Prognostic Value of Neutrophil-to-Lymphocyte Ratio in Patients with Acute Decompensated Heart Failure: A Meta-Analysis. J. Clin. Med..

[28] Zhang J.L., Yang R., Zhu Y., Shao Y., Ji Y., Wang F.F. (2023). Association between the neutrophil-to-lymphocyte ratio and risk of in-hospital heart failure and arrhythmia in patients with acute myocardial infarction. Front. Cardiovasc. Med..

[29] Davison B.A., Takagi K., Edwards C., Adams K.F., Butler J., Collins S.P., Dorobantu M.I., Ezekowitz J.A., Filippatos G., Greenberg B.H. (2022). Neutrophil-to-Lymphocyte Ratio and Outcomes in Patients Admitted for Acute Heart Failure (As Seen in the BLAST-AHF, Pre-RELAX-AHF, and RELAX-AHF Studies). Am. J. Cardiol..

[30] Cakir M.O. (2023). The Prognostic Significance of Neutrophil-to-Lymphocyte Ratio and Platelet-to-Lymphocyte Ratio for Long-Term Survival in Patients with Severe Left Ventricular Dysfunction and Implantable Cardioverter Defibrillator. Cureus.

[31] Benites-Zapata V.A., Hernandez A.V., Nagarajan V., Cauthen C.A., Starling R.C., Tang W.H. (2015). Usefulness of neutrophil-to-lymphocyte ratio in risk stratification of patients with advanced heart failure. Am. J. Cardiol..

[32] Sadeghi M.T., Esgandarian I., Nouri-Vaskeh M., Golmohammadi A., Rahvar N., Teimourizad A. (2020). Role of circulatory leukocyte based indices in short-term mortality of patients with heart failure with reduced ejection fraction. Med. Pharm. Rep..

[33] Seropian I.M., Romeo F.J., Pizarro R., Vulcano N.O., Posatini R.A., Marenchino R.G., Berrocal D.H., Belziti C.A. (2018). Neutrophil-to-lymphocyte ratio and platelet-to-lymphocyte ratio as predictors of survival after heart transplantation. ESC Heart Fail..

[34] Bhatt D.L., Lopes R.D., Harrington R.A. (2022). Diagnosis and Treatment of Acute Coronary Syndromes: A Review. JAMA.

[35] Thayabaranathan T., Kim J., Cadilhac D.A., Thrift A.G., Donnan G.A., Howard G., Howard V.J., Rothwell P.M., Feigin V., Norrving B. (2022). Global stroke statistics 2022. Int. J. Stroke.

[36] Ong S.B., Hernández-Reséndiz S., Crespo-Avilan G.E., Mukhametshina R.T., Kwek X.Y., Cabrera-Fuentes H.A., Hausenloy D.J. (2018). Inflammation following acute myocardial infarction: Multiple players, dynamic roles, and novel therapeutic opportunities. Pharmacol. Ther..

[37] Arruda-Olson A.M., Reeder G.S., Bell M.R., Weston S.A., Roger V.L. (2009). Neutrophilia predicts death and heart failure after myocardial infarction: A community-based study. Circ. Cardiovasc. Qual. Outcomes.

[38] Arbel Y., Shacham Y., Ziv-Baran T., Laufer Perl M., Finkelstein A., Halkin A., Revivo M., Milwidsky A., Berliner S., Herz I. (2014). Higher neutrophil/lymphocyte ratio is related to lower ejection fraction and higher long-term all-cause mortality in ST-elevation myocardial infarction patients. Can. J. Cardiol..

[39] Shahsanaei F., Abbaszadeh S., Behrooj S., Rahimi Petrudi N., Ramezani B. (2024). The value of neutrophil-to-lymphocyte ratio in predicting severity of coronary involvement and long-term outcome of percutaneous coronary intervention in patients with acute coronary syndrome: A systematic review and meta-analysis. Egypt. Heart J..

[40] Pruc M., Kubica J., Banach M., Swieczkowski D., Rafique Z., Peacock W.F., Siudak Z., Kurek K., Nanayakkara P., Szarpak L. (2024). Diagnostic and prognostic performance of the neutrophil-to-lymphocyte ratio in acute coronary syndromes: A meta-analysis of 90 studies including 45,990 patients. Kardiol. Pol..

[41] Fedrizal F.F., Wijaya I.P., Abdullah M., Yamin M. (2024). Elevated neutrophyl-to-lymphocyte ratioand smoking are associated with chronic total occlusion in patients with ST elevation myocardial infarction. BMC Cardiovasc. Disord..

[42] Oncel R.C., Ucar M., Karakas M.S., Akdemir B., Yanikoglu A., Gulcan A.R., Altekin R.E., Demir I. (2015). Relation of neutrophil-to-lymphocyte ratio with GRACE risk score to in-hospital cardiac events in patients with ST-segment elevated myocardial infarction. Clin. Appl. Thromb. Hemost..

[43] Ji Z., Liu G., Guo J., Zhang R., Su Y., Carvalho A., Qu Y., Zuo W., Yao Y., Lin J. (2021). The Neutrophil-to-Lymphocyte Ratio Is an Important Indicator Predicting In-Hospital Death in AMI Patients. Front. Cardiovasc. Med..

[44] Maleki M., Tajlil A., Separham A., Sohrabi B., Pourafkari L., Roshanravan N., Aslanabadi N., Najjarian F., Mashayekhi S., Ghaffari S. (2021). Association of neutrophil to lymphocyte ratio (NLR) with angiographic SYNTAX score in patients with non-ST-Segment elevation acute coronary syndrome (NSTE-ACS). J. Cardiovasc. Thorac. Res..

[45] Chen Y., Chen S., Han Y., Xu Q., Zheng M., Zhao X. (2023). A combined index constructed based on NLR and PLR is associated with in-hospital mortality risk in patients with acute myocardial infarction. Am. J. Transl. Res..

[46] Verdoia M., Nardin M., Gioscia R., Negro F., Marcolongo M., Suryapranata H., Kedhi E., De Luca G. (2020). Higher neutrophil-to-lymphocyte ratio (NLR) increases the risk of suboptimal platelet inhibition and major cardiovascular ischemic events among ACS patients receiving dual antiplatelet therapy with ticagrelor. Vasc. Pharmacol..

[47] Xu N., Tang X.F., Yao Y., Zhao X., Chen J., Gao Z., Yang Y., Gao R.L., Xu B., Yuan J.Q. (2018). Predictive value of neutrophil to lymphocyte ratio in long-term outcomes of left main and/or three-vessel disease in patients with acute myocardial infarction. Catheter. Cardiovasc. Interv..

[48] Akpek M., Kaya M.G., Lam Y.Y., Sahin O., Elcik D., Celik T., Ergin A., Gibson C.M. (2012). Relation of neutrophil/lymphocyte ratio to coronary flow to in-hospital major adverse cardiac events in patients with ST-elevated myocardial infarction undergoing primary coronary intervention. Am. J. Cardiol..

[49] Pinheiro Machado G., Araujo G.N., Carpes C.K., Lech M.C., Mariani S., Valle F.H., Bergoli L.C.C., Wainstein R.V., Wainstein M.V. (2019). Elevated neutrophil-to-lymphocyte ratio can predict procedural adverse events in patients with ST-elevation myocardial infarction undergoing primary percutaneous coronary intervention. Coron. Artery Dis..

[50] Wang L., Huang S., Zhou Q., Dou L., Lin D. (2024). The predictive value of laboratory parameters for no-reflow phenomenon in patients with ST-elevation myocardial infarction following primary percutaneous coronary intervention: A meta-analysis. Clin. Cardiol..

[51] Zhang Q., Hu M., Sun J., Ma S. (2020). The combination of neutrophil-to-lymphocyte ratio and platelet correlation parameters in predicting the no-reflow phenomenon after primary percutaneous coronary intervention in patients with ST-segment elevation myocardial infarction. Scand. Cardiovasc. J..

[52] Del Turco S., Basta G., De Caterina A.R., Sbrana S., Paradossi U., Taddei A., Trianni G., Ravani M., Palmieri C., Berti S. (2019). Different inflammatory profile in young and elderly STEMI patients undergoing primary percutaneous coronary intervention (PPCI): Its influence on no-reflow and mortality. Int. J. Cardiol..

[53] Caimi G., Lo Presti R., Canino B., Ferrera E., Hopps E. (2016). Behaviour of the neutrophil to lymphocyte ratio in young subjects with acute myocardial infarction. Clin. Hemorheol. Microcirc..

[54] Chen B., Yuan L., Chen X., Li J., Tao J., Li W., Zheng R. (2020). Correlations and Prognostic Roles of the Nutritional Status and Neutrophil-to-lymphocyte Ratio in Elderly Patients with Acute Myocardial Infarction Undergoing Primary Coronary Intervention. Int. Heart J..

[55] Dentali F., Nigro O., Squizzato A., Gianni M., Zuretti F., Grandi A.M., Guasti L. (2018). Impact of neutrophils to lymphocytes ratio on major clinical outcomes in patients with acute coronary syndromes: A systematic review and meta-analysis of the literature. Int. J. Cardiol..

[56] Chen Y., Chen S., Han Y., Xu Q., Zhao X. (2023). Neutrophil-to-Lymphocyte Ratio and Platelet-to-Lymphocyte Ratio are Important Indicators for Predicting in-Hospital Death in Elderly AMI Patients. J. Inflamm. Res..

[57] Zhang S., Diao J., Qi C., Jin J., Li L., Gao X., Gong L., Wu W. (2018). Predictive value of neutrophil to lymphocyte ratio in patients with acute ST segment elevation myocardial infarction after percutaneous coronary intervention: A meta-analysis. BMC Cardiovasc. Disord..

[58] Chen C., Cong B.L., Wang M., Abdullah M., Wang X.L., Zhang Y.H., Xu S.J., Cui L. (2018). Neutrophil to lymphocyte ratio as a predictor of myocardial damage and cardiac dysfunction in acute coronary syndrome patients. Integr. Med. Res..

[59] Fan W., Zhang Y., Gao X., Liu Y., Shi F., Liu J., Sun L. (2021). The Prognostic Value of a Derived Neutrophil-Lymphocyte Ratio in Patients with Acute Coronary Syndrome Undergoing Percutaneous Coronary Intervention. Clin. Appl. Thromb. Hemost..

[60] Wang X., Wei C., Fan W., Sun L., Zhang Y., Sun Q., Liu Y., Liu J. (2023). Advanced Lung Cancer Inflammation Index for Predicting Prognostic Risk for Patients with Acute Coronary Syndrome Undergoing Percutaneous Coronary Intervention. J. Inflamm. Res..

[61] Rostami A., Tajlil A., Separham A., Sohrabi B., Pourafkari L., Roshanravan N., Aslanabadi N., Ziaee M., Mashayekhi S., Ghaffari S. (2021). Association between Neutrophil-to-Lymphocyte Ratio and the Systemic Inflammatory Immunologic Index and the Angiographic SYNTAX Score and the TIMI Flow Grade in Acute STEMI: A Cohort Study. J. Tehran Heart Cent..

[62] Ha E.T., Yee A., Peterson S.J., Kobayashi Y., Sacchi T., Parikh M., Brener S.J. (2024). Neutrophil-to-lymphocyte ratio and prognosis in patients undergoing percutaneous coronary intervention for acute coronary syndrome. Cardiovasc. Revasc Med..

[63] Awada M., Sanaei S., Jameie M., Rahnamoun Z. (2024). Effects of cardiac rehabilitation on inflammatory biomarkers in unstable ischemic heart disease patients following percutaneous coronary intervention: A randomized controlled study. Coron. Artery Dis..

[64] Tudurachi B.S., Anghel L., Tudurachi A., Sascău R.A., Stătescu C. (2023). Assessment of Inflammatory Hematological Ratios (NLR, PLR, MLR, LMR and Monocyte/HDL-Cholesterol Ratio) in Acute Myocardial Infarction and Particularities in Young Patients. Int. J. Mol. Sci..

[65] Luo J., Thomassen J.Q., Nordestgaard B.G., Tybjærg-Hansen A., Frikke-Schmidt R. (2023). Neutrophil counts and cardiovascular disease. Eur. Heart J..

[66] Li B., Lai X., Yan C., Jia X., Li Y. (2020). The associations between neutrophil-to-lymphocyte ratio and the Chinese Visceral Adiposity Index, and carotid atherosclerosis and atherosclerotic cardiovascular disease risk. Exp. Gerontol..

[67] Wang X., Chen X., Wang Y., Peng S., Pi J., Yue J., Meng Q., Liu J., Zheng L., Chan P. (2023). The Association of Lipoprotein(a) and Neutrophil-to-Lymphocyte Ratio Combination with Atherosclerotic Cardiovascular Disease in Chinese Patients. Int. J. Gen. Med..

[68] Li X., Li J., Wu G. (2021). Relationship of Neutrophil-to-Lymphocyte Ratio with Carotid Plaque Vulnerability and Occurrence of Vulnerable Carotid Plaque in Patients with Acute Ischemic Stroke. Biomed. Res. Int..

[69] Corriere T., Di Marca S., Cataudella E., Pulvirenti A., Alaimo S., Stancanelli B., Malatino L. (2018). Neutrophil-to-Lymphocyte Ratio is a strong predictor of atherosclerotic carotid plaques in older adults. Nutr. Metab. Cardiovasc. Dis..

[70] Ruan W., Wang M., Sun C., Yao J., Ma Y., Ma H., Ding J., Lian X. (2022). Correlation between neutrophil-to-lymphocyte ratio and stability of carotid plaques. Clin. Neurol. Neurosurg..

[71] (2018). Global, regional, and national age-sex-specific mortality for 282 causes of death in 195 countries and territories, 1980–2017: A systematic analysis for the Global Burden of Disease Study 2017. Lancet.

[72] Bagyura Z., Kiss L., Lux A., Csobay-Novak C., Jermendy A.L., Polgar L., Tabak A.G., Soos P., Szelid Z., Merkely B. (2023). Neutrophil-to-Lymphocyte Ratio Is an Independent Risk Factor for Coronary Artery Disease in Central Obesity. Int. J. Mol. Sci..

[73] Tangjitgamol S., Udayachalerm W., Wanishsawad C., Kaewwanna W., Ativanichayapong N. (2024). Association of Neutrophil-to-Lymphocyte Ratio and Platelet-to-Lymphocyte Ratio and Coronary Artery Disease Among the Physicians. J. Inflamm. Res..

[74] Xu X., Zhu X., Wang H., Liu X., Yang C., Liu L., Chen T., Cai L., Zhu H. (2024). Evaluation of the Prognostic Role of Neutrophil-Lymphocyte Ratio, C-Reactive Protein-Albumin Ratio, and Platelet-Lymphocyte Ratio in Patients with the Co-Presentation of Coronary Artery Disease and COVID-19. Infect. Drug Resist..

[75] Wada H., Dohi T., Miyauchi K., Nishio R., Takeuchi M., Takahashi N., Endo H., Ogita M., Iwata H., Kasai T. (2020). Neutrophil to Lymphocyte Ratio and Long-Term Cardiovascular Outcomes in Coronary Artery Disease Patients with Low High-Sensitivity C-Reactive Protein Level. Int. Heart J..

[76] Larmann J., Handke J., Scholz A.S., Dehne S., Arens C., Gillmann H.J., Uhle F., Motsch J., Weigand M.A., Janssen H. (2020). Preoperative neutrophil to lymphocyte ratio and platelet to lymphocyte ratio are associated with major adverse cardiovascular and cerebrovascular events in coronary heart disease patients undergoing non-cardiac surgery. BMC Cardiovasc. Disord..

[77] Shah N., Parikh V., Patel N., Patel N., Badheka A., Deshmukh A., Rathod A., Lafferty J. (2014). Neutrophil lymphocyte ratio significantly improves the Framingham risk score in prediction of coronary heart disease mortality: Insights from the National Health and Nutrition Examination Survey-III. Int. J. Cardiol..

[78] Wang Q.C., Wang Z.Y. (2023). Comparative analysis of neutrophil-to-lymphocyte ratio and remnant cholesterol in predicting cardiovascular events and mortality in general adult population. Sci. Rep..

[79] Verma R., Moroney M., Hibino M., Mazer C.D., Connelly K.A., Yan A.T., Quan A., Teoh H., Verma S., Puar P. (2023). Baseline neutrophil-to-lymphocyte ratio and efficacy of SGLT2 inhibition with empagliflozin on cardiac remodelling. ESC Heart Fail..

[80] Higaki A., Caillon A., Paradis P., Schiffrin E.L. (2019). Innate and Innate-Like Immune System in Hypertension and Vascular Injury. Curr. Hypertens. Rep..

[81] Tsuda K. (2024). A link between white blood cell count and blood pressure levels. Hypertens. Res..

[82] Mansoori A., Farizani Gohari N.S., Etemad L., Poudineh M., Ahari R.K., Mohammadyari F., Azami M., Rad E.S., Ferns G., Esmaily H. (2024). White blood cell and platelet distribution widths are associated with hypertension: Data mining approaches. Hypertens. Res..

[83] Krishnan J., Hennen E.M., Ao M., Kirabo A., Ahmad T., de la Visitación N., Patrick D.M. (2024). NETosis Drives Blood Pressure Elevation and Vascular Dysfunction in Hypertension. Circ. Res..

[84] Kılıçaslan B., Dursun H., Kaymak S., Aydın M., Ekmekçi C., Susam İ., Özdoğan Ö. (2015). The relationship between neutrophil to lymphocyte ratio and blood pressure variability in hypertensive and normotensive subjecs. Turk. Kardiyol. Dern. Ars..

[85] Drugescu A., Roca M., Zota I.M., Costache A.D., Leon-Constantin M.M., Gavril O.I., Gavril R.S., Vasilcu T.F., Mitu O., Ghiciuc C.M. (2023). Relationships between Easily Available Biomarkers and Non-Dipper Blood Pressure Pattern in Patients with Stable Coronary Artery Disease. Life.

[86] Sarejloo S., Dehesh M., Fathi M., Khanzadeh M., Lucke-Wold B., Ghaedi A., Khanzadeh S. (2023). Meta-analysis of differences in neutrophil to lymphocyte ratio between hypertensive and non-hypertensive individuals. BMC Cardiovasc. Disord..

[87] Sunbul M., Gerin F., Durmus E., Kivrak T., Sari I., Tigen K., Cincin A. (2014). Neutrophil to lymphocyte and platelet to lymphocyte ratio in patients with dipper versus non-dipper hypertension. Clin. Exp. Hypertens..

[88] Yu X., Xue Y., Bian B., Wu X., Wang Z., Huang J., Huang L., Sun Y. (2020). NLR-A Simple Indicator of Inflammation for the Diagnosis of Left Ventricular Hypertrophy in Patients with Hypertension. Int. Heart J..

[89] Sun X., Luo L., Zhao X., Ye P., Du R. (2017). The neutrophil-to-lymphocyte ratio on admission is a good predictor for all-cause mortality in hypertensive patients over 80 years of age. BMC Cardiovasc. Disord..

[90] Karagöz A., Vural A., Günaydın Z.Y., Bektaş O., Gül M., Çelik A., Uzunoğlu E., Usta S., Sarıtaş A., Elalmış Ö.U. (2015). The role of neutrophil to lymphocyte ratio as a predictor of diastolic dysfunction in hypertensive patients. Eur. Rev. Med. Pharmacol. Sci..

[91] Gao X., Jiang X., Wu Z., Chen N., Gong M., Zhao X., Liu Y., Guo R. (2024). Effect of Neutrophil-to-Lymphocyte Ratio on Post-TAVR Mortality and Periprocedural Pulmonary Hypertension. J. Interv. Cardiol..

[92] Du P., Gao X., Sun Q., Gong M., Pan Y., Guo Q., Zhao X., Guo R., Liu Y. (2024). Association between uric acid and cardiac outcomes mediated by neutrophil-to-lymphocyte ratio in patients with left ventricular diastolic dysfunction and pulmonary hypertension. Sci. Rep..

[93] Skrzypczyk P., Zacharzewska A., Szyszka M., Ofiara A., Panczyk-Tomaszewska M. (2021). Arterial stiffness in children with primary hypertension is related to subclinical inflammation. Cent. Eur. J. Immunol..

[94] Hindricks G., Potpara T., Dagres N., Arbelo E., Bax J.J., Blomström-Lundqvist C., Boriani G., Castella M., Dan G.A., Dilaveris P.E. (2021). Corrigendum to: 2020 ESC Guidelines for the diagnosis and management of atrial fibrillation developed in collaboration with the European Association for Cardio-Thoracic Surgery (EACTS): The Task Force for the diagnosis and management of atrial fibrillation of the European Society of Cardiology (ESC) Developed with the special contribution of the European Heart Rhythm Association (EHRA) of the ESC. Eur. Heart J..

[95] Al-Khatib S.M. (2023). Atrial Fibrillation. Ann. Intern. Med..

[96] Burdett P., Lip G.Y.H. (2022). Atrial fibrillation in the UK: Predicting costs of an emerging epidemic recognizing and forecasting the cost drivers of atrial fibrillation-related costs. Eur. Heart J. Qual. Care Clin. Outcomes.

[97] Ihara K., Sasano T. (2022). Role of Inflammation in the Pathogenesis of Atrial Fibrillation. Front. Physiol..

[98] Fagundes A., Ruff C.T., Morrow D.A., Murphy S.A., Palazzolo M.G., Chen C.Z., Jarolim P., Antman E.M., Braunwald E., Giugliano R.P. (2023). Neutrophil-lymphocyte ratio and clinical outcomes in 19,697 patients with atrial fibrillation: Analyses from ENGAGE AF- TIMI 48 trial. Int. J. Cardiol..

[99] Li Q., Nie J., Cao M., Luo C., Sun C. (2024). Association between inflammation markers and all-cause mortality in critical ill patients with atrial fibrillation: Analysis of the Multi-Parameter Intelligent Monitoring in Intensive Care (MIMIC-IV) database. Int. J. Cardiol. Heart Vasc..

[100] Magoon R., Shri I., Kashav R.C., Dey S., Kohli J.K., Grover V., Gupta V. (2023). Atrial Fibrillation and Perioperative Inflammation (FIBRILLAMMED Study): A Retrospective Analysis of the Predictive Role of Preoperative Albumin-Adjusted Platelet-Leukocytic Indices in OPCABG. Turk. J. Anaesthesiol. Reanim..

[101] Boyalla V., Harling L., Snell A., Kralj-Hans I., Barradas-Pires A., Haldar S., Khan H.R., Cleland J.G.F., Athanasiou T., Harding S.E. (2022). Biomarkers as predictors of recurrence of atrial fibrillation post ablation: An updated and expanded systematic review and meta-analysis. Clin. Res. Cardiol..

[102] Ding B., Liu P., Zhang F., Hui J., He L. (2022). Predicting Values of Neutrophil-to-Lymphocyte Ratio (NLR), High-Sensitivity C-Reactive Protein (hs-CRP), and Left Atrial Diameter (LAD) in Patients with Nonvalvular Atrial Fibrillation Recurrence After Radiofrequency Ablation. Med. Sci. Monit..

[103] Rizza V., Maranta F., Cianfanelli L., Cartella I., Maisano F., Alfieri O., Cianflone D. (2024). Subacute postoperative atrial fibrillation after heart surgery: Incidence and predictive factors in cardiac rehabilitation. J. Arrhythm..

[104] Liu Z., Nguyen Khuong J., Borg Caruana C., Jackson S.M., Campbell R., Ramson D.M., Penny-Dimri J.C., Kluger M., Segal R., Perry L.A. (2020). The Prognostic Value of Elevated Perioperative Neutrophil-Lymphocyte Ratio in Predicting Postoperative Atrial Fibrillation After Cardiac Surgery: A Systematic Review and Meta-Analysis. Heart Lung Circ..

[105] Jacob K.A., Buijsrogge M.P., Frencken J.F., Ten Berg M.J., Suyker W.J., van Dijk D., Dieleman J.M. (2017). White blood cell count and new-onset atrial fibrillation after cardiac surgery. Int. J. Cardiol..

[106] Baba D.F., Suciu H., Avram C., Gyorgy M., Danilesco A., Huma L., Sin I.A. (2023). Elevated Levels of Neutrophil-to Monocyte Ratio Are Associated with the Initiation of Paroxysmal Documented Atrial Fibrillation in the First Two Months after Heart Transplantation: A Uni-Institutional Retrospective Study. J. Cardiovasc. Dev. Dis..

[107] Mirna M., Schmutzler L., Topf A., Hoppe U.C., Lichtenauer M. (2021). Neutrophil-to-lymphocyte ratio and monocyte-to-lymphocyte ratio predict length of hospital stay in myocarditis. Sci. Rep..

[108] Vahanian A., Beyersdorf F., Praz F., Milojevic M., Baldus S., Bauersachs J., Capodanno D., Conradi L., De Bonis M., De Paulis R. (2021). 2021 ESC/EACTS Guidelines for the management of valvular heart disease: Developed by the Task Force for the management of valvular heart disease of the European Society of Cardiology (ESC) and the European Association for Cardio-Thoracic Surgery (EACTS). Eur. Heart J..

[109] Otto C.M., Nishimura R.A., Bonow R.O., Carabello B.A., Erwin J.P., Gentile F., Jneid H., Krieger E.V., Mack M., McLeod C. (2021). 2020 ACC/AHA Guideline for the Management of Patients with Valvular Heart Disease: A Report of the American College of Cardiology/American Heart Association Joint Committee on Clinical Practice Guidelines. Circulation.

[110] Cho K.I., Sakuma I., Sohn I.S., Jo S.H., Koh K.K. (2018). Inflammatory and metabolic mechanisms underlying the calcific aortic valve disease. Atherosclerosis.

[111] Varol E., Aksoy F., Ozaydin M., Erdogan D., Dogan A. (2014). Association between neutrophil-lymphocyte ratio and mitral annular calcification. Blood Coagul. Fibrinolysis.

[112] Yayla Ç., Akboga M.K., Canpolat U., Gayretli Yayla K., Kuyumcu M.S., Bayraktar F., Suleymanoglu M., Aydogdu S. (2015). The association of the platelet-to-lymphocyte ratio with mitral annular calcification. Scand. Cardiovasc. J..

[113] Küçükseymen S., Çağırcı G., Güven R., Arslan Ş. (2017). Is neutrophyl to lymphocyte ratio really a useful marker for all grades of degenerative aortic stenosis?. Turk. Kardiyol. Dern. Ars..

[114] Tian H., Jiang X., Duan G., Chen J., Liu Q., Zhang Y., Li S., Bao X., Huang H. (2023). Preoperative inflammatory markers predict postoperative clinical outcomes in patients undergoing heart valve surgery: A large-sample retrospective study. Front. Immunol..

[115] Song J., Zheng Q., Ma X., Zhang Q., Xu Z., Zou C., Wang Z. (2019). Predictive Roles of Neutrophil-to-Lymphocyte Ratio and C-Reactive Protein in Patients with Calcific Aortic Valve Disease. Int. Heart J..

[116] Lindman B.R., Goldstein J.S., Nassif M.E., Zajarias A., Novak E., Tibrewala A., Vatterott A.M., Lawler C., Damiano R.J., Moon M.R. (2015). Systemic inflammatory response syndrome after transcatheter or surgical aortic valve replacement. Heart.

[117] Abu Khadija H., Gandelman G., Ayyad O., Jaber M., Poles L., Jonas M., Paz O., Abu Sbaih F., Sella G., Shimoni S. (2021). Differential systemic inflammatory responses after TAVI: The role of self versus balloon expandable devices. PLoS ONE.

[118] Habib M., Thawabi M., Hawatmeh A., Habib H., ElKhalili W., Shamoon F., Zaher M. (2018). Value of neutrophil to lymphocyte ratio as a predictor of mortality in patients undergoing aortic valve replacement. Cardiovasc. Diagn. Ther..

[119] Condado J.F., Junpaparp P., Binongo J.N., Lasanajak Y., Witzke-Sanz C.F., Devireddy C., Leshnower B., Mavromatis K., Stewart J., Guyton R. (2016). Neutrophil-lymphocyte ratio (NLR) and platelet-lymphocyte ratio (PLR) can risk stratify patients in transcatheter aortic-valve replacement (TAVR). Int. J. Cardiol..

[120] Li P., Xia C., Liu P., Peng Z., Huang H., Wu J., He Z. (2020). Neutrophil-to-lymphocyte ratio and platelet-to-lymphocyte ratio in evaluation of inflammation in non-dialysis patients with end-stage renal disease (ESRD). BMC Nephrol..

[121] Kaya M.G., Akpek M., Lam Y.Y., Yarlioglues M., Celik T., Gunebakmaz O., Duran M., Ulucan S., Keser A., Oguzhan A. (2013). Prognostic value of neutrophil/lymphocyte ratio in patients with ST-elevated myocardial infarction undergoing primary coronary intervention: A prospective, multicenter study. Int. J. Cardiol..

[122] Lee M.J., Park S.D., Kwon S.W., Woo S.I., Lee M.D., Shin S.H., Kim D.H., Kwan J., Park K.S. (2016). Relation Between Neutrophil-to-Lymphocyte Ratio and Index of Microcirculatory Resistance in Patients with ST-Segment Elevation Myocardial Infarction Undergoing Primary Percutaneous Coronary Intervention. Am. J. Cardiol..

[123] Shahim B., Redfors B., Lindman B.R., Chen S., Dahlen T., Nazif T., Kapadia S., Gertz Z.M., Crowley A.C., Li D. (2022). Neutrophil-to-Lymphocyte Ratios in Patients Undergoing Aortic Valve Replacement: The PARTNER Trials and Registries. J. Am. Heart Assoc..

[124] Afari M.E., Bhat T. (2016). Neutrophil to lymphocyte ratio (NLR) and cardiovascular diseases: An update. Expert. Rev. Cardiovasc. Ther..

[125] Aarts C.E.M., Kuijpers T.W. (2018). Neutrophils as myeloid-derived suppressor cells. Eur. J. Clin. Investig..

[126] Ge Y., Cheng D., Jia Q., Xiong H., Zhang J. (2021). Mechanisms Underlying the Role of Myeloid-Derived Suppressor Cells in Clinical Diseases: Good or Bad. Immune Netw..

[127] Zhang M., Shi X., Zhao J., Guo W., Zhou J. (2023). Recruitment of myeloid-derived suppressor cells and regulatory T-cells is associated with the occurrence of acute myocardial infarction. Biomed. Rep..

[128] Wang Y.G., Xiong X., Chen Z.Y., Liu K.L., Yang J.H., Wen Q., Wu F.Q., Hu X.F., Peng Y.D., Wu J.J. (2015). Expansion of myeloid-derived suppressor cells in patients with acute coronary syndrome. Cell Physiol. Biochem..

[129] Sweetnam P.M., Thomas H.F., Yarnell J.W., Baker I.A., Elwood P.C. (1997). Total and differential leukocyte counts as predictors of ischemic heart disease: The Caerphilly and Speedwell studies. Am. J. Epidemiol..

[130] Huang Z.S., Chien K.L., Yang C.Y., Wang C.H., Chang T.C., Chen C.J. (2003). Peripheral differential leukocyte counts and subsequent mortality from all diseases, cancers, and cardiovascular diseases in Taiwanese. J. Formos. Med. Assoc..

[131] Angkananard T., Inthanoo T., Sricholwattana S., Rattanajaruskul N., Wongsoasu A., Roongsangmanoon W. (2021). The Predictive role of Neutrophil-to-Lymphocyte Ratio (NLR) and Mean Platelet Volume-to-Lymphocyte Ratio (MPVLR) for Cardiovascular Events in Adult Patients with Acute Heart Failure. Mediat. Inflamm..

[132] Shao Q., Chen K., Rha S.W., Lim H.E., Li G., Liu T. (2015). Usefulness of Neutrophil/Lymphocyte Ratio as a Predictor of Atrial Fibrillation: A Meta-analysis. Arch. Med. Res..

[133] Vakhshoori M., Nemati S., Sabouhi S., Yavari B., Shakarami M., Bondariyan N., Emami S.A., Shafie D. (2023). Neutrophil to lymphocyte ratio (NLR) prognostic effects on heart failure; a systematic review and meta-analysis. BMC Cardiovasc. Disord..

[134] Adamstein N.H., MacFadyen J.G., Rose L.M., Glynn R.J., Dey A.K., Libby P., Tabas I.A., Mehta N.N., Ridker P.M. (2021). The neutrophil-lymphocyte ratio and incident atherosclerotic events: Analyses from five contemporary randomized trials. Eur. Heart J..

